# Hypoglycemic effect of aqueous extract of *Trichosanthes dioica* in normal and diabetic rats

**DOI:** 10.4103/0973-3930.60011

**Published:** 2010

**Authors:** Shalini Adiga, K. L. Bairy, A. Meharban, I. S. R. Punita

**Affiliations:** Department of Pharmacology, Kasturba Medical College, Manipal - 576 104, India

**Keywords:** Diabetic rats, hypoglycemic effect, *Trichosanthes dioica*

## Abstract

**Background:**

*Trichosanthes dioica* is used to treat diabetes mellitus, epilepsy, alopecia, and skin disease in folklore medicine. The leaf extract of the plant is used in diabetes mellitus but there have been no scientific studies reported.

**Aims:**

To study the effect of *Trichosanthes dioica* on serum glucose level in glucose loaded, normal and hyperglycemic rats.

**Settings:**

Kasturba Medical College, Manipal, Karnataka, India.

**Design:**

Experimental.

**Materials and Methods:**

The aqueous extract of leaves of *Trichosanthes dioica* are compared with glibeclamide for their influence on fasting blood sugar in glucose loaded, normoglycemic and streptozotocin induced (45 mg/kg ip) hyperglycemic rats.

**Statistical Analysis:**

The data was analyzed by one way ANOVA followed by Scheffe's post hoc test.

**Results:**

In glucose loaded rats, normal rats and hyperglycemic rats the aqueous extract at both the doses (800 mg/kg/p.o and 1600 mg/kg/p.o) reduced blood glucose significantly when compared to control but it was not as effective as glibenclamide.

**Conclusion:**

The aqueous extract of *Trichosanthes dioica* has antihyperglycemic action.

## Introduction

In India, Ayurvedic medicine is reported to have been successfully used in the treatment of diabetes mellitus.[5] As an alternative mode of treatment, Ayurvedic medicine has been claimed to be less toxic and more efficacious. In accordance to the recommendations of World Health Organization (WHO) Expert committee on diabetes mellitus, an investigation of antihyperglycemic agents of plant origin used in traditional medicine seems important.[6] Many herbs and plant products have shown to have antihyperglycemic effect.[7–11]

In indigenous system of medicines, *Trichosanthes dioica* (*T*. *dioica*, parwal or pointed gourd) is used to treat epilepsy, alopecia, skin disease and diabetes mellitus.[12] Rai *et al*. have reported that the LD_50_ of *T*. *dioica* is above 15 g/kg and the plant extract has shown significant reduction in liver enzymes (Alanine transaminase and Alkaline phosphatase) and serum creatinine.[13] The fruits and seeds of the plant have been reported to have hypoglycemic activity.[14,15] The leaves of the plant are used in diabetes mellitus but there are no scientific studies on its hypoglycemic activity. This study aims to evaluate the antihyperglycemic activity of aqueous extract of leaves of *T*. *dioica* in normal and streptozotocin induced diabetic rats.

## Materials and Methods

### Animals

Male albino rats (200-250 g body weight), raised in the Central Animal House of Manipal University, have been used in the study. They were fed on standard chow and given tap water *ad libitum*. The study was approved by the Institutional Animal Ethics Committee. Animal care and handling was done as per the guidelines set by the Indian National Science Academy New Delhi, India. Animals described as fasted were deprived of food for 16 hours but had free access to water.

A total of 96 rats were segregated into 16 groups of six animals each. Four groups were used for glucose tolerance tests. Another eight groups were used to study hypoglycemic activity in normal rats. The remaining four groups were used to study the drug effects in diabetic rats.

### Preparation of plant extract

The leaves of *T*. *dioica* were collected from a local shop and voucher specimen was deposited in Botany Laboratory of Mahatma Gandhi Memorial College, Udupi, India after botanical identification. The dried leaves were crushed into moderately coarse powder. A kilogram of the coarse powder was immersed in distilled water in a flask for seven days. The solid residue obtained by straining the liquid was pressed and filtered. The filtrate was concentrated on water bath to get a viscous paste.[16] It was finally dried in a dessicator. The yield was 4.25%.

#### Glucose tolerance tests

Fasted rats were divided into four groups of six animals each. Group I served as control and received distilled water. Group II and III received aqueous extract at an oral dose of 800 and 1600 mg/kg respectively. Group IV received the standard drug glibenclamide 0.5 mg/kg orally.

All the groups were loaded with 50% glucose (2 g/kg/p.o) 30 minutes after drug administration.[17] Blood samples were collected from the tail vein just prior to drug administration and at 30, 60, 120 minutes after glucose loading. Serum glucose levels were measured immediately.

#### Hypoglycemic study in normal fasted rats

The effect of aqueous extract on fasting blood glucose was studied in normal rats. Animals were divided into eight groups of six rats each. Four groups received single dose of either distilled water, plant extract (800 mg and 1600 mg/ kg) or glibenclamide 0.5 mg/kg. The blood was collected at 30, 60 and 120 minutes after drug administration, to estimate blood sugar. The remaining four groups received the drug as above for 15 days. Fasting blood sample was collected on day 0,7,15 and 30 for estimating blood sugar.

#### Induction of diabetes

Streptozotocin (STZ) obtained from Sigma chemicals Co, was dissolved in 0.9% ice cold saline immediately before use. The rats were made to fast overnight, administered STZ 45 mg/kg intraperitoneally.[18] Fasting blood glucose levels were determined on day 12 after administering STZ to confirm stable hyperglycemia.

### Determination of efficacy of aqueous extract of *T. dioica* in diabetic rats

The STZ induced diabetic rats were divided into four groups of six each. Group 1 received distilled water and served as control. Group II and III received aqueous extract of *T. dioica* at a dose 800 and 1600 mg/kg/p.o respectively. Group IV received the standard drug glibenclamide 0.5 mg/kg. The treatment was continued for 15 days. Fasting blood samples were collected on days 0, 7 and 15 of drug administration. Food and water intake was monitored daily for each rat during 15 days of experimental period.

### Statistical analysis

Results are expressed as mean ± SE. The data was analyzed by one way ANOVA followed by Scheffe's post hoc test using SPSS computer package. *P* < 0.05 were considered significant.

## Results

Effects of the extract on glucose loaded rats are shown in Table 1. It is observed that the treatment of *T*. *dioica* leaf extract at both the doses of 800 mg/kg and 1600 mg/ kg reduced blood sugar significantly as observed at 30, 60 and 120 minutes when compared to normal control rats. Similarly, Glibenclamide treated rats also showed significant blood glucose lowering efficacy at all the time intervals.

**Table 1:** Effect of aqueous extract from *Trichosanthes dioica* on blood glucose levels in glucose loaded hyperglycemic (OGTT) rats

Groups (n)	Dose (mg/kg)	Fasting blood sugar in mg/dl			
		
		0 min	30 min	60 min	120 min
Control	-	72.25 ± 6.52	116.50 ± 4.09	114.25 ± 2.78	105.75 ± 5.39
Aqueous extract	800	74.50 ± 3.57	83.00 ± 6.40*	91.50 ± 2.90*	81.00 ± 5.68*
Aqueous extract	1600	90.75 ± 6.62	98.75 ± 2.75*	91.25 ± 3.06*	92.75 ± 2.42
Glibenclamide	0.5	76.25 ± 4.17	103.25 ± 1.60	88.50 ± 2.36*	81.00 ± 3.31*

One way ANOVA F 12.2, 16.98, 7.11; df 3, 12 3, 12 3, 12; *P* < 0.05 < 0.05 < 0.05. Values expressed as mean ± S.E.M; n = 6 in each group;

* *P* < 0.05 significant as compared to control, df = 3, 12

Administration of the plant extract was found to reduce blood glucose level in normoglycemic rats both in single dose and repeated dose administration for 15 days [Tables 2 and 3]. In a single dose study the maximum reduction in blood glucose was noted at one hour for 800 mg/kg, 30 minutes for 1600 mg/kg and the standard drug, glibenclamide. In the multidose study, the treated rats (extract/drug) showed significantly reduced blood glucose levels on days 7, 15 and the values returned to the pretreatment levels by day 30.

**Table 2:** Effect of single dose administration of *Trichosanthes dioica* aqueous extract on blood glucose levels in normal rats

Groups (n)	Dose (mg/kg)	Fasting blood sugar in mg/dl			
		
		0 min	30 min	60 min	120 min
Control	-	65.50 ± 2.02	64.50 ± 1.65	72.25 ± 3.94	80.00 ± 4.56
Aqueous extract	800	63.00 ± 2.67	60.00 ± 2.94	58.50 ± 3.17*	61.50 ± 2.21*
Aqueous extract	1600	62.00 ± 2.51	49.50 ± 2.10*	51.50 ± 1.19*	62.00 ± 1.08*
Glibenclamide	0.5	66.00 ± 3.10	54.00 ± 2.16*	52.25 ± 2.39*	66.75 ± 2.68*

One way ANOVA F 8.4, 11.2, 8.7; df 3,12 3,12 3,12; *P* < 0.05 < 0.05 < 0.05. Values expressed as Mean ± S.E.M; n = 6 in each group;

* *P* < 0.05 significant as compared to control, df = 3, 12

**Table 3:** Effect of repeated administration of *Trichosanthes dioica* aqueous extract on blood glucose levels in normal rats

Groups (n)	Dose (mg/kg)	Fasting blood sugar (FBS) in mg/dl			
		
		Day 0	Day 7	Day 15	Day 30
Control	-	65.50 ± 2.02	76.00 ± 4.41	75.50 ± 3.79	81.75 ± 4.11
Aqueous extract	800	63.00 ± 2.67	56.25 ± 2.17**	50.25 ± 2.78**	62.00 ± 2.16*
Aqueous extract	1600	62.00 ± 2.51	56.50 ± 1.70**	51.75 ± 1.18**	66.75 ± 1.18*
Glibenclamide	0.5	66.00 ± 3.16	60.75 ± 1.49**	55.75 ± 1.70**	68.50 ± 1.32*

One way ANOVA F 18.8, 20.6, 11.5; df 3,12 3,12 3,12; *P* < 0.05 < 0.05 < 0.05. Values expressed as Mean ± S.E.M; n = 6 in each group;

* *P* < 0.05;

** *P* < 0.001 significant as compared to control, df = 3, 12

The pretreatment fasting blood glucose levels in STZ induced diabetic rats were 250-260 mg/dl. After continuous administration of the extracts, the glycemic levels were found to decrease significantly from 251 mg/ dl to 213 mg/dl (800 mg/dl), 218 mg/dl (1600 mg/ kg) on 7^th^ day, and to 177 mg/dl (800 mg/dl), 172 mg/dl (1600 mg/ kg) on 15^th^ day [Table 4].

**Table 4:** Effect of repeated administration of *Trichosanthes dioica* aqueous extract on blood glucose levels in diabetic rats

Groups (n)	Dose (mg/kg)	Fasting blood suga (FBS) in mg/dl		
		
		Day 0	Day 7	Day 15
Control	-	259.33 ± 3.99	262.33 ± 2.81	286.50 ± 1.91
Aqueous extract	800	251.00 ± 2.16	213.33 ± 2.57*	177.66 ± 4.21*
Aqueous extract	1600	251.66 ± 3.81	218.16 ± 3.68*	172.33 ± 2.91*
Glibenclamide	0.5	255.83 ± 2.07	184.33 ± 5.53*	113.66 ± 5.35*

One way ANOVA F 70.59, 354.17; df 3,20 3,20; *P* < 0.001 < 0.001. Values expressed as Mean ± S.E.M, n = 6 in each group;

* *P* < 0.001 significant as compared to control, df = 3, 20

The Glibenclamide treated diabetic rats also showed a significant reduction from 255 mg/dl to 184.33 on day 7 and to 113.66 on day 15. Though the hypoglycemic activity of the extract treated rats were significant when compared to control it was not as effective as glibenclamide.

The effects of *T*. *dioica* extract on the body weight changes of diabetic rats are shown in Table 5. During the two weeks of observation of the treated diabetic rats there was weight loss, relative to day 0, i.e. before the start of the treatment. The untreated diabetic rats lost 16.38% of their body weight. The loss was 9.75% and 8% (*P* < 0.05) for 800 mg/kg and1600 mg/kg respectively, significantly lower as compared to control. The diabetic rats treated with Glibenclamide also showed a bodyweight reduction of 5.5%, which is significantly much lesser than control diabetic rats. The untreated diabetic rats had severe polyphagia and polydipsia by the end of week 2 of experiment with respective increase in food and fluid intakes of 43 and 20%. However, in the presence of extract the food intake was reduced significantly to 35% by day 7 and 51% by day 15(*P* < 0.05) with 800 mg/kg and 34% in week 1, 41% (*P* < 0.05) in week 2 with 1600 mg/kg as compared with diabetic control rats. A similar reduction of 37% in week 1 and 57% in week 2 was observed for glibenclamide, the standard drug [Table 6].

**Table 5:** Body weight changes in diabetic treated rats

Groups (n)	Dose (mg/kg)	Day 0	Day 7	Day 15
Control	-	219.83 ± 2.85	201.83 ± 2.35	183.83 ± 2.28
Aqueous extract	800	215.33 ± 3.71	205.00 ± 3.37	194.33 ± 2.26*
Aqueous extract	1600	213.66 ± 3.84	204.50 ± 3.95	196.50 ± 2.41*
Glibenclamide	0.5	209.66 ± 3.72	201.83 ± 3.91	198.00 ± 1.87*

One way ANOVA F 0.24, 8.3; df 3, 20 3, 20; *P* < 0.86 < 0.001. Values expressed as Mean ± S.E.M, n = 6 in each group;

* Significant as compared to control, df = 3, 20

**Table 6:** Food intake in diabetic treated rats

Groups (n)	Dose (mg/kg)	Food intak (g/rat/week)		
		
		Day 0	Week 1	Week 2
Control	-	16.5 ± 1.20	22.66 ± 0.55	23.66 ± 0.42
Aqueous extract	800	15.33 ± 1.02	9.83 ± 0.30*	7.50 ± 0.42*
Aqueous extract	1600	16.16 ± 0.65	10.66 ± 0.33*	9.50 ± 0.61*
Glibenclamide	0.5	16.16 ± 0.94	10.16 ± 0.30*	6.83 ± 0.42*

One way ANOVA F 254.1, 278.6; df 3, 20 3, 20; *P* < 0.001 < 0.001. Values expressed as Mean ± S.E.M, n = 6 in each group;

* Significant as compared to control, df = 3, 20

Fluid intake decreased by 32% (*P* < 0.05) in rats treated with both doses of *T*. *dioica*. Diabetic rats treated with glibenclamide also showed a significant lower water intake of 48% (*P* < 0.05) as compared to control [Table 7].

**Table 7:** Water intake in diabetic treated rats

Groups (n)	Dose (mg/kg)	Water intake (ml/rat/week)		
		
		Day 0	Week 1	Week 2
Control	-	54.50 ± 4.27	60.83 ± 2.81	65.16 ± 2.83
Aqueous extract	800	63.00 ± 1.98	51.00 ± 1.80	42.66 ± 1.92*
Aqueous extract	1600	61.83 ± 1.83	57.16 ± 1.64	41.83 ± 2.24*
Glibenclamide	0.5	66.66 ± 2.77	50.33 ± 2.53*	34.33 ± 1.64*

One way ANOVA F 4.4, 36.4; df 3, 20, 3, 20; *P* < 0.015 < 0.001 n = 6, values expressed as Mean ± S.E.M;

* Significant as compared to control, df = 3, 20

## Discussion

Diabetes is a chronic disease affecting millions of people world wide. The WHO expert committee has aptly suggested that research should be aimed at investigating the traditional methods of treatment for refractory diseases like diabetes.[19]

Our study has detected the antidiabetic activity of aqueous extract of *T*. *dioica* in STZ induced type 2 diabetic rats. STZ is a valuable tool in the experimental production of non insulin dependent diabetes mellitus (NIDDM) model in lab animals. The mechanisms by which STZ brings about its diabetic state include selective destruction of pancreatic insulin secreting beta cells, which make cells less active.[20] The use of lower dose of STZ (45 mg/kg) produced an incomplete destruction of pancreatic beta cells even though rats became permanently diabetic.[21] The glucose loaded model indicates the kinetic functional changes induced by any antidiabetic drug.

When *T*. *dioica* aqueous leaves extract was administered to glucose-loaded normal rats, hypoglycemia was observed after 30 minutes, with maximum effects being seen at two hours. Our results also indicate the efficacy of aqueous extract in the maintenance of glucose levels in normal and streptozotocin induced diabetic rats. However, it was less efficacious than glibenclamide. It is well established that sulphonylureas cause hypoglycemia by stimulating insulin release from pancreatic beta cells.[22] The comparable effect of the extract with glibenclamide suggests the possibility of a similar mode of action.

Induction of diabetes with streptozotocin is associated with a characteristic loss of body weight, which is probably due to muscle wasting.[23] In our study there was a significant weight loss in the vehicle treated diabetic rats, where as treatment with the aqueous extract of leaves of *T*. *dioica* at both the doses showed improvement in their body weight, indicating that the aqueous extract had beneficial effect in preventing loss of body weight of diabetic rats [Table 5]. The probable mechanism of this benefit is due to its effect in controlling muscle wasting, i.e., by reversal of antagonism.[24] The metabolic disturbances were corrected after the plant extract was administered for two weeks as shown by a reduction in polyphagia, polyuria and polydipsia in diabetic rats treated with plant extract.

## Conclusion

The aqueous extract of the leaves of *T*. *dioica* has antidiabetic activity as it lowers serum glucose levels in diabetic rats and significantly increases glucose tolerance. It also increases body weight of diabetic rats. Further studies are necessary to substantiate the above observation and to work out the exact mechanism of action involved in the antidiabetic activity of this plant.
